# Additional sampling directions improve detection range of wireless radiofrequency probes

**DOI:** 10.1002/mrm.25993

**Published:** 2015-09-29

**Authors:** Malte Hoffmann, Marius Mada, T. Adrian Carpenter, Stephen J. Sawiak, Guy B. Williams

**Affiliations:** ^1^ Wolfson Brain Imaging Centre, Department of Clinical NeurosciencesUniversity of CambridgeCambridgeUnited Kingdom; ^2^ Behavioural and Clinical Neuroscience Institute, University of CambridgeCambridgeUnited Kingdom

**Keywords:** wireless RF markers, head motion, prospective correction

## Abstract

**Purpose:**

While MRI is enhancing our knowledge about the structure and function of the human brain, subject motion remains a problem in many clinical applications. Recently, the use of wireless radiofrequency markers with three one‐dimensional (1D) navigators for prospective correction was demonstrated. This method is restricted in the range of motion that can be corrected, however, because of limited information in the 1D readouts.

**Methods:**

Here, the limitation of techniques for disambiguating marker locations was investigated. It was shown that including more sampling directions extends the tracking range for head rotations. The efficiency of trading readout resolution for speed was explored.

**Results:**

Tracking of head rotations was demonstrated from −19.2 to 34.4°, −2.7 to 10.0°, and −60.9 to 70.9° in the x‐, y‐, and z‐directions, respectively. In the presence of excessive head motion, the deviation of marker estimates from SPM8 was reduced by 17.1% over existing three‐projection methods. This was achieved by using an additional seven directions, extending the time needed for readouts by a factor of 3.3. Much of this increase may be circumvented by reducing resolution, without compromising accuracy.

**Conclusion:**

Including additional sampling directions extends the range in which markers can be used, for patients who move a lot. Magn Reson Med 76:913–918, 2016. © 2015 The Authors. Magnetic Resonance in Medicine published by Wiley Periodicals, Inc. on behalf of International Society for Magnetic Resonance in Medicine. This is an open access article under the terms of the Creative Commons Attribution License, which permits use, distribution and reproduction in any medium, provided the original work is properly cited.

## INTRODUCTION

MRI scanners allow increasingly high‐resolution imaging to be performed. Yet acquisitions remain limited by sensitivity to motion, which causes artefacts. Various methods have been proposed to correct motion retrospectively 1, 2, 3, 4, 5 or prospectively 6, 7, 8, 9, 10, 11. We recently showed that there are limits on how much activation retrospective techniques can recover in fMRI 12. Prospective methods allow updates to be applied during the data collection, reducing spin‐history effects that are otherwise difficult to address 13, 14. Recently, the use of wireless radiofrequency markers 15 for prospective correction was demonstrated 16, 17 using three one‐dimensional (1D) navigator readouts before the imaging sequence. This provides a rapid method for locating points in the volume of interest that can be used for motion correction.

The signal from each probe appears as a peak at its projected location in the tracking data. From these projections alone, it is not possible to uniquely determine which peak results from which marker [the “correspondence problem” 17].

If the set of probes rotates too far from its original orientation, peaks can overlap or cross, making motion tracking difficult. Additionally, the orientation of the probes relative to B0 affects their signal, ideally the probe axis will be as close to perpendicular to B0 as possible. Ooi et al attached three markers to a 3D‐printed spectacles frame 17 fixing the relative positions of the probes in space (hereinafter referred to as the “glasses” method). Within a range of rotations, the peaks do not overlap. Translations are not a problem, as they cause peaks to shift in the same way. If a patient cannot be examined with the head in the standard position, it is possible that peaks overlap after minimal rotations.

With the assumptions by Sengupta et al 16 the markers could represent the vertices of 36 different triangles. By comparing their side lengths with the first acquisition (for which the correspondence is known), the correct triangle can be inferred and marker locations determined (hereinafter referred to as the “triangles” method). Constraints on the derived parameters are used to regularize the fitting process 16. However, problems can be observed when peaks overlap, and the initial marker arrangement needs to be known to derive motion in the correct space.

We previously characterized the motion of patients in disorders of consciousness during a functional MRI (fMRI) checkerboard paradigm 12. For these patients we observed particularly large motion during a motor imagery task (imagining playing tennis): 5.2% of 96 patients performed head rotations in excess of 17° in the z‐direction. For our setup, such large motion caused existing marker methods to fail.

Here, we investigate the practical limitation of the “glasses” and “triangles” methods. We present a method that increases the number of 1D readouts to disambiguate the marker locations (hereinafter referred to as the “directions” method). This back projection was first proposed by Ooi et al 17 and has the advantage that a greater range of rotations can be resolved such as may need to be tracked in the most challenging clinical populations. To compare against retrospective volume‐based registration, we acquired marker projections but did not apply the motion parameters during the data acquisition, although the presented algorithm is sufficiently rapid to allow this.

We investigated the limitation imposed by the need to align each probe as close as possible to the perpendicular of B0, to assess what rotations are tolerable for our algorithms. To speed up the navigator, we compared the error on motion estimates for a range of sampling resolutions.

## METHODS

### Marker Construction and Tracking

Wireless markers were made following 17 for use on a three‐Tesla (T) MR system (Siemens Healthcare, Erlangen, Germany). Marker positions were measured using the pulse sequence described by 16. Each 1D readout took 5 ms [field of view (FOV) 600 mm, 1024 sample points]. In addition to the x‐, y‐, and z‐axes, we acquired readouts along two further sets of directions, evenly distributed on the surface of a sphere when passing through its center (the first with six and the second with 12 directions, see Supporting Table S1, which is available online).

Peaks were identified as described in Sengupta et al 16. Only peaks above a threshold of 10 standard deviations (SD) of the background signals were accepted.

### RF Safety Testing

In common with previous uses of wireless markers 16 we assessed safety by measuring temperature changes using a fiber‐optic thermometer (LumaSense Technologies, Santa Clara, CA) during three sequences: (a) low flip angle 3D GRE, repetition time/echo time (TR/TE) 51/2.1 ms, FA 10°, NA 20, TA 13 min 21 s, (b) high flip angle 3D GRE, TR/TE 51/2.1 ms, FA 90°, NA 20, TA 13 min 21 s, (c) 3D FSE, TR/TE 1160/56 ms, FA 90/150°, ETL 32, NA 10, TA 15 min 31 s. To test the effect of a high SAR sequence, we performed an additional 3D‐FSE experiment (TR/TE 1070/7.6 ms, FA 90/180°, ETL 128, NA 10, TA 15 min 44 s). The SAR for a 60‐kg patient reported by the scanner for each of these sequences was 3, 20, 33, and 100% of the SAR limit, respectively. Furthermore, we used the manufacturer‐provided B1 mapping protocol to produce flip angle maps with and without the probes. All experiments were performed at 3T.

### Maximum Signal Detection Range

The signal from one marker was measured along 21 directions as a function of the angle between its axis and B0. Two bags of saline solution were placed 15 cm left/right to the marker along the x‐axis. Peaks along each direction were identified as the maximum value in the projections, and normalized to the highest peak along that direction.

### Coordinate Correspondence

We determined the marker locations choosing the first three noncollinear readout directions **d** *_i_* (*i* = 1,2,3) along which we detected three peaks *p_ij_* (*j* = 1,2,3). Each peak defines a plane E*_ij_* orthogonal to **d** *_i_*. We computed the points of intersection **m** *_n_* (*n*=1,2,…,27) between all combinations of E*_ij_*. To find the marker locations, we used the remaining directions **d** *_k_* (*k*=4,5,…,*N*) and orthogonal planes E*_kj_*. The distance of each point **m** *_n_* to each of these planes was transformed into a score using a Gaussian function of unit height and 2.3 mm full width at half maximum (FWHM) (FWHM of the peaks in the projections); the values were summed over *j* and *k* to give a metric that ranked the points **m** *_n_* in terms of their likelihood of being the marker positions.

**Figure 1:**
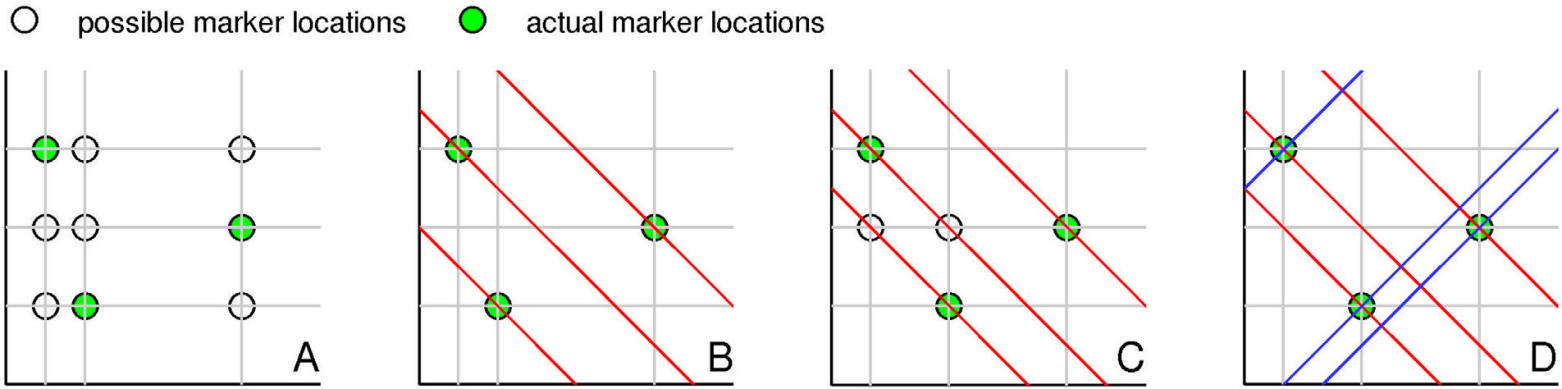
Two‐dimensional schematic showing how more sampling directions reduce the ambiguity in the back projection. **A**: Two directions with three markers could represent nine locations. **B**: A third direction removes the ambiguity and uniquely identifies the probes. **C**: A slightly different configuration, however, is still ambiguous. **D**: A fourth direction resolves the issue. In practice, a statistical approach is needed, as described in the text. [Color figure can be viewed in the online issue, which is available at wileyonlinelibrary.com.]

A 2D example of how the additional navigators are used is shown in Figure 1. Because the marker locations must be consistent with each acquired signal, increasing the number of navigators reduces ambiguities that may result.

When three peaks were detected along fewer than six of the 21 directions, it was not possible to determine the marker locations, and tracking data were interpolated by copying estimates from the previous acquisition

### Comparison of Motion Estimates In Vivo

We compared wireless markers to retrospective registration of echo planar imaging (EPI) using SPM8 1. A volunteer was instructed to move his head systematically, and scanned with the navigator prepended to the acquisition of every EPI volume (TR/TE 2020/18 ms, matrix 96 × 96, FOV 300 × 300 mm^2^, 31 3‐mm slices with 1.5 mm gap). The coordinates were right‐handed (i.e., x left–right, y posterior–anterior, z inferior–superior) for a patient lying supine. The study was approved by the Cambridgeshire 2 Research Ethics committee (02/293).

We calculated position estimates with the “glasses,” “triangles,” and “directions” methods offline, and compared them with registration. For each frame, the side lengths of the triangle defined by the estimated marker locations were compared. In case of deviations from the reference exceeding 3% of the total length, the estimation was discarded and parameters were interpolated.

An algorithm was developed to improve probe labeling using prior knowledge. Assuming that the two minimally separated peaks in the previous dataset are most likely to overlap, the peak closest to their mean location was considered twice, if only two peaks were detected. Hereinafter, we will denote methods using this algorithm with an asterisk.

We calculated the “RMS deviation” between transformation matrices derived using markers and SPM8 when applied to a point and integrated over the brain (128‐mm sphere) 18. Maximum displacements at the surface of the brain were calculated by converting rotations into translations according to Tisdall et al 19.

### Navigator Resolution

We simulated faster navigator acquisition by downsampling the acquired data by a factor of *n* (*n* = 1.14, 1.33, 1.60, 2.00) using linear interpolation. Motion parameters were derived and compared with those estimated by SPM8.

## RESULTS

### RF Safety Testing

We found similar results to previous safety tests 16. The probes did not heat up by more than 0.5°C compared with the reference temperature. B1 mapping of a phantom with and without probes showed that the markers caused a maximum change of 1.3%.

### Maximum Signal Detection Range

**Figure 2:**
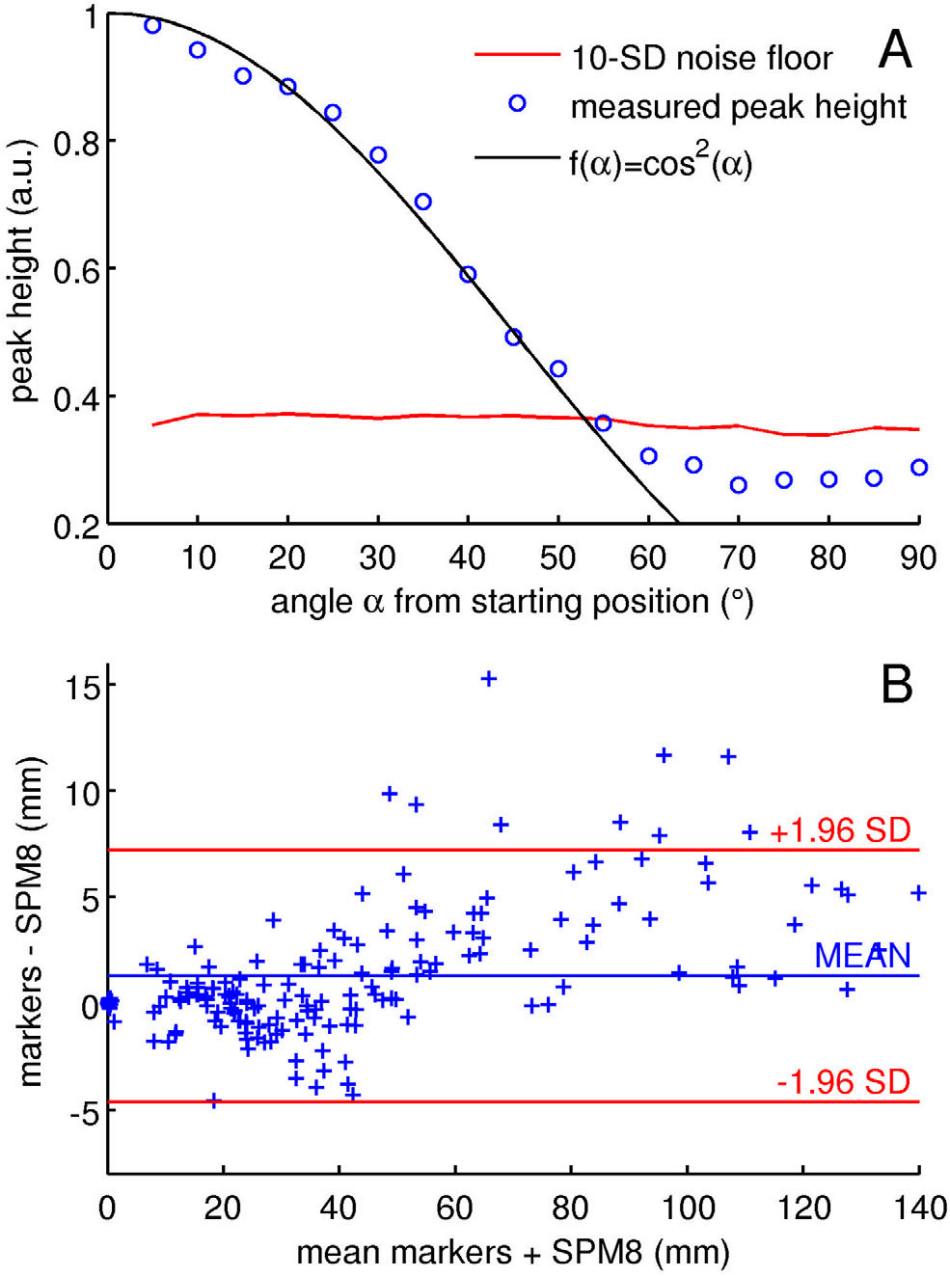
**A**: Peak height with regard to the angle between the marker axis and the left–right axis (for rotations in the x–z plane). In the starting position the probe axis was perpendicular to B0 (measurements started at 85° from B0). The red line marks the noise level used for peak detection, defined as 10 standard deviations of the background signal in the navigator data. Shown are mean values over 21 readout directions. The theoretical signal is plotted in black. **B**: Comparison between the maximum motion derived from SPM8 and markers using additional sampling directions (mean 1.1 mm, standard deviation 3.3 mm). [Color figure can be viewed in the online issue, which is available at wileyonlinelibrary.com.]

The average peak height resulting from one marker is shown in Figure 2A for increasing alignment with B0. In the starting position the probe and x‐axis were parallel. Signal strength decreased until it dropped to below the noise floor used for peak detection. This happened at ∼55°, resulting in a theoretical detection range of approximately 55° between the x‐axis and B0.

### Comparison of Motion Estimates In Vivo

**Figure 3:**
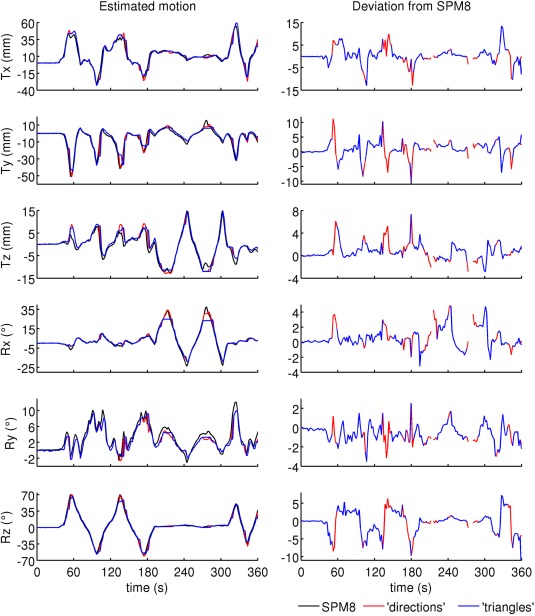
Motion estimates from image registration of EPI (SPM8), wireless markers using additional sampling directions (“directions”) and the method by Sengupta et al (“triangles”). Deviations from SPM8 are shown for points where motion tracking did not fail. Marker positions were determined with the “directions” algorithm. Minimum/maximum motion estimates derived from the probes were (relative to the starting position): $T_{x}$ −33.4/61.0 mm, $T_{y}$ −51.5/10.9 mm, $T_{z}$ −13.0/14.6 mm, $R_{x}$ −19.2/34.4°, $R_{y}$ −2.7/10.0°, $R_{z}$ −60.9/70.9°. [Color figure can be viewed in the online issue, which is available at wileyonlinelibrary.com.]

The best performing methods (“directions” and “triangles”) are compared in Figure 3. We found that peak overlap confounded the “glasses” method for rotations outside the ranges $-16.6{}^{\circ}\leq R_{x}\leq 21.2{}^{\circ}$ and $\;-17.0{}^{\circ}\leq R_{z}\leq 30.2{}^{\circ}$.

Table 1 shows the discrepancies between SPM8 and probe measurements. When estimating overlapping peak positions, the mean RMS deviation of the “glasses” and “triangles” methods was reduced by 8.1% and 13.3%, respectively. Performance of the “directions” method could not be improved: the mean RMS deviation increased by 4.6%. In some cases, peaks were below the noise level (due to low angles between the marker axis and B0), so that incorrect peak locations were being allocated.

**Table 1:** Mean RMS Deviation (mm) from SPM8 and Number of Frames (Out of 180) at Which the Estimation Was Successful or Failed (Either Due to Incorrect Probe Locations or an Insufficient Number of Detected Peaks)^a^

	Frames with correctly labeled probes		Frames with incorrectly labeled probes		Probe detection failure		All frames
Method	Number	RMS deviation	Number	RMS deviation	Number	RMS deviation	RMS deviation
Directions	176	4.09	0	‐	4	8.05	4.18
Directions*	169	4.13	11	8.04	0	‐	4.37
Glasses	110	2.70	46	22.73	24	18.07	9.87
Glasses*	118	2.79	59	21.25	3	16.52	9.07
Triangles	149	3.76	7	6.60	24	12.60	5.04
Triangles*	164	4.00	13	6.76	3	14.48	4.37

a Methods marked with an asterisk make use of the adapted algorithm considering overlapping peak locations. When motion tracking failed, parameters were interpolated by copying estimates from the previous time point.

While the average deviation from SPM8 was of the order of a voxel size for correctly labeled points, we measured a value of 0.2 ± 0.1 mm for all methods during the first 30 s, when the subject did not move deliberately.

At times the angle of the markers was too small with respect to B0 (∼210/270 s in Figure 3). The resulting signal drop caused an insufficient number of peaks to be identified. Throughout the rest of the time course, three peaks were detected along at least 11 directions (mean 16.7).

Maximum displacements at the surface of the brain for SPM8 and the “directions” method are compared in Figure 2B. The estimates from SPM8 were shifted by half a TR to compensate for half the time it takes to acquire an image. Frames for which motion correction failed were excluded.

### Readout Resolution

Table 2 summarizes the performance of the two best performing methods (“directions” and “triangles*”) at different resolutions. When using the “directions” method, RMS deviation did not change significantly for down to 384 sampling points. The “triangles*” method was less robust at lower resolutions.

**Table 2:** Change in Mean RMS Deviation from SPM8 (in mm) and Number of Frames Out of 180 at Which the Estimation Failed for Different Readout Resolutions (Either Due to Insufficient Signal or Incorrect Probe Locations)^a^

		‘Directions’			‘Triangles*’		
Sampling points	Resolution (mm)	Change in RMS deviation	Failure due to insufficient signal	Failure due to misidentified probe locations	Change in RMS deviation	Failure due to insufficient signal	Failure due to misidentified probe locations
1024	0.59	0.00	4	0	0.00	13	3
896	0.67	−0.05	4	0	0.03	14	3
768	0.78	−0.05	4	0	0.15	15	3
640	0.94	−0.03	4	0	0.18	16	1
512	1.17	−0.06	4	0	0.35	17	4
384	1.57	−0.05	4	0	0.85	28	4
256	2.35	0.23	6	1	1.36	36	10

a Use of overlapping peak estimation is marked with an asterisk.

## DISCUSSION

Wireless markers have been shown to accurately determine motion. We have shown here that with our spectacles frame the limits of the approach by Ooi et al 17 are −16.6/21.2° and −17.0/30.2° for rotations about the x‐ and z‐axes, respectively. These limits may be exceeded in patient populations prone to motion. The “triangles” method 16 extends the range of rotations covered. However, problems can be observed when peaks overlap.

Here, we show that including more directions extends the tracking range to any head rotation for arbitrary initial positions unless peaks fade due to alignment with B0. This may allow a greater number of successful scans. We demonstrated tracking in the ranges of $‐19.2{}^{\circ}\leq R_{x}\leq 34.4{}^{\circ}$ and $\;‐60.9{}^{\circ}\leq R_{z}\leq 70.9{}^{\circ}$, and extended the theoretical rotational tracking range to ±55° about the x‐axis (for markers aligned with the y‐axis).

A similar experiment characterizing the marker signal as a function of the angle between the marker axis and B0 was performed in Sengupta et al 16. The signal was found to decrease similarly, and presented as a function of the angle with the left–right and anterior–posterior axes. Here, peak heights were averaged over 21 directions to find a threshold for peak identification. While over‐flipping during RF transmit could cause signal drops below the noise floor, here, the signal was lost predictably with the orientation of the probes with B0.

On average, RMS deviations of about a voxel size were measured for frames with correctly assigned probe locations. These differences are driven by frames with large motion: when the subject did not move deliberately, the mean RMS deviation was 0.2 ± 0.1 mm for all methods. Large deviations could be due to the EPI volumes encoding the mean motion during one TR. In contrast, marker readouts occur almost instantaneously. If the subject moves in one direction, marker estimates represent instant motion amplitudes instead of motion averaged over the acquisition of the volume. It is, therefore, difficult to describe image registration as a “gold standard.”

Part of the deviations from SPM8 are due to a delay in the EPI estimates. When the estimates were shifted by half a TR to compensate for half the time it takes to acquire an image, the match between probe data and SPM8 was improved in Figure 3. The RMS deviation for frames with correctly labeled probes in Table 1 decreased by 29.1–35.0%. The ranking of the performance of each method, however, was not affected. Similarly, the range of maximum motion differences in Figure 2B was reduced from 0.8 ± 6.3 to 1.1 ± 3.3 mm.

Although 21 directions were acquired, the method gave identical results when only the first 10 were used. Overall, this required 35 ms/TR more than the “triangles” and “glasses” methods. To reduce the time taken for each readout, we showed that the tracking accuracy of the new algorithm is not affected for readouts of down to 37.5% of the resolution used here. Acquiring fewer samples in the same time (i.e., at a lower bandwidth) may also improve the SNR of the navigator data.

Resolution could not be decreased as much without compromising tracking accuracy with the “triangles*” algorithm. Due to the lack of redundancy in the data, failure to detect sufficient peaks resulted in interpolation and increased RMS deviation. The ability of choosing complete over incomplete readouts made the “directions” method more robust. Further work is needed to exploit the redundancy in the data to improve tracking accuracy.

Estimating locations of overlapping peaks, we improved the performance of existing methods by 13.3% in the presence of exceptional motion. Using seven additional directions, motion estimates were improved by further 3.8%. For small ranges of motion which do not cause any peaks to overlap, all methods output identical motion parameters (the “triangles” method failed 1.9% of the time in simulation, however 16).

Processing the navigator data (180 frames, 1024 sampling points, 21 directions) with the “directions” method took 4.5 s in MATLAB (MathWorks, Natick, MA) on a 2.4‐GHz workstation. This results in 25 ms/TR, and should be short enough for prospective correction with TRs of 1–2 s. In practice, using a compiled routine will reduce the processing time. Acquiring 10 signals took 50 ms, but this could be shortened to 19 ms by reducing the readout resolution.

The marker signal can appear as bright spots in the image for similar navigator/imaging TEs 17. These spots, however, appear outside the anatomy of interest. Other methods such as vNavs 19 and fatNavs 20, 21 measure motion without additional hardware, but require more sophisticated pulse sequences and longer acquisition times of 275 ms and 1.15 s 20, respectively. It is possible that large motion might dislodge the markers, but we did not experience this in vigorous volunteer testing. A detailed comparison of these methods together has not yet been performed and is an important next step.

While EPI data were used to derive motion parameters in this work, methods based on wireless markers are applicable to acquisitions which segment k‐space; volume‐based registration cannot be used in conjunction with these techniques.

## CONCLUSIONS

Our algorithm extends the tracking range of markers to any head rotation unless the signal fades due to alignment with B0, for use in patients who move a lot. We demonstrated tracking of exceptional head rotations from −19.2 to 34.4°, −2.7 to 10.0°, and −60.9 to 70.9° in the x‐, y‐, and z‐direction, respectively. We improved the performance of wireless‐marker–based motion tracking by 17.1% for excessive rotations. This was achieved using data from an additional seven readout directions, which extends the time needed for navigator readouts by a factor of 3.3. We showed that much of this increase in acquisition time may be circumvented by decreasing resolution, without compromising accuracy. Our method extends the utility of wireless markers to patient populations where there may be more extreme motion.

## Supporting information


**Supporting Table S1**. Directions along which the marker signal was sampled (in the scanner coordinate system). In addition to the x‐, y‐, and z‐axes, readouts were acquired along two further sets of directions (21 directions in total), chosen such that they were evenly distributed on the surface of a sphere when passing through its center (the first with six directions and the second with 12 directions).
