# Supplementary material for: Additional sampling directions improve detection range of wireless radiofrequency probes
Source: Magn Reson Med. 2015 Sep 29;76(3):913–8. doi: 10.1002/mrm.25993 (PMC5025722; doi:10.1002/mrm.25993)
Supplement: Supplementary file 1 — Supporting Table S1. Directions along which the marker signal was sampled (in the scanner coordinate system). In addition to the x‐, y‐, and z‐axes, readouts were acquired along two further sets of directions (21 directions in total), chosen such that they were evenly distributed on the surface of a sphere when passing through its center (the first with six directions and the second with 12 directions). [file MRM-76-913-s001.docx]

**Additional tables**

*Supporting Table S1*

Directions along which the marker signal was sampled (in the scanner coordinate system). In addition to the x-, y- and z-axes, readouts were acquired along two further sets of directions (21 directions in total), chosen such that they were evenly distributed on the surface of a sphere when passing through its centre (the first with six directions and the second with 12 directions).

| **Index** | **Sampling direction** |
| --- | --- |
| 1 | (1, 0, 0 ) |
| 2 | (0, 1, 0 ) |
| 3 | (0, 0, 1 ) |
| 4 | (0.7071, 0, 0.7071 ) |
| 5 | (-0.7071, 0, 0.7071 ) |
| 6 | (0, -0.7071, 0.7071 ) |
| 7 | (0, -0.7071, -0.7071) |
| 8 | (0.7071, -0.7071, 0 ) |
| 9 | (-0.7071, -0.7071, 0 ) |
| 10 | (0.8628, -0.3574, -0.3574) |
| 11 | (0.8628, 0.3574, -0.3574) |
| 12 | (0.8628, 0.3574, 0.3574 ) |
| 13 | (0.8628, -0.3574, 0.3574 ) |
| 14 | (0.3574, -0.3574, 0.8628 ) |
| 15 | (0.3574, -0.8628, 0.3574 ) |
| 16 | (0.3574, -0.8628, -0.3574) |
| 17 | (0.3574, -0.3574, -0.8628) |
| 18 | (0.3574, 0.3574, -0.8628) |
| 19 | (0.3574, 0.8628, -0.3574) |
| 20 | (0.3574, 0.8628, 0.3574) |
| 21 | (0.3574, 0.3574, 0.8628) |
